# DUSP12 Regulates NAT10‐Mediated RNA Acetylation to Modulate DNA Repair and Therapeutic Response in Hepatocellular Carcinoma

**DOI:** 10.1002/mco2.70929

**Published:** 2026-08-17

**Authors:** Viktor Kalbermatter Boell, Diana Reis Della Corte Guimarães Pacheco, Yuli Thamires Magalhães, Laura Lima Turani, Isabeli Yumi Araújo Osawa, Nicolas Carlos Hoch, Fábio Luís Forti

**Affiliations:** ^1^ Department of Biochemistry Institute of Chemistry University of São Paulo (USP) São Paulo Brazil

**Keywords:** doxorubicin, dual‐specificity phosphatase 12 (DUSP12), hepatocellular carcinoma (HCC), N4‐acetylcytidine (ac4C), *N*‐acetyltransferase 10 (NAT10), nucleolar stress

## Abstract

Hepatocellular carcinoma (HCC), an aggressive type of liver cancer, has limited treatment options, and chemotherapy remains an important clinical approach. This study investigates the role of dual‐specificity phosphatase 12 (DUSP12) and its interaction with the nucleolar protein *N*‐acetyltransferase 10 (NAT10) in HCC models under genotoxic stress. We demonstrate that doxorubicin (DX) induces greater cytotoxicity than cisplatin, correlating with a stronger DNA damage response (DDR), nucleolar stress, and NAT10 relocalization. CRISPR‐Cas9‐mediated DUSP12 knockout sensitized cells to DX, increasing markers of DNA damage (γH2AX, p53) and delaying DNA break repair. This was accompanied by redistribution of nucleolar proteins NAT10 and TCOF1. Remodelin treatment enhanced DX sensitivity in DUSP12 knockout cells, suggesting a context‐dependent interaction between DUSP12 loss and the NAT10 inhibition. Protein interaction assays confirmed that DUSP12 binds NAT10, corroborating a regulatory interaction. DUSP12 deletion increased NAT10 phosphotyrosine levels and reduced N4‐acetylcytidine (ac4C) RNA modification, consistent with altered NAT10‐dependent RNA acetylation. Analysis of patient data revealed frequent *DUSP12* amplification in HCC, whereas elevated DUSP12 expression was associated with poor survival and enrichment of DDR‐ and ribosome biogenesis‐related transcriptional programs. Our findings identify the DUSP12–NAT10–ac4C axis as a novel molecular link between DDR and nucleolar stress, unveiling a potential therapeutic vulnerability in HCC.

## Graphical abstract

In hepatocellular carcinoma (HCC) cells exposed to doxorubicin, DUSP12 depletion maintains NAT10 phosphorylation, reducing its acetyltransferase activity, which leads to reduced ac4C RNA acetylation, DNA damage response, and DNA break repair, with consequent tumor sensitization.

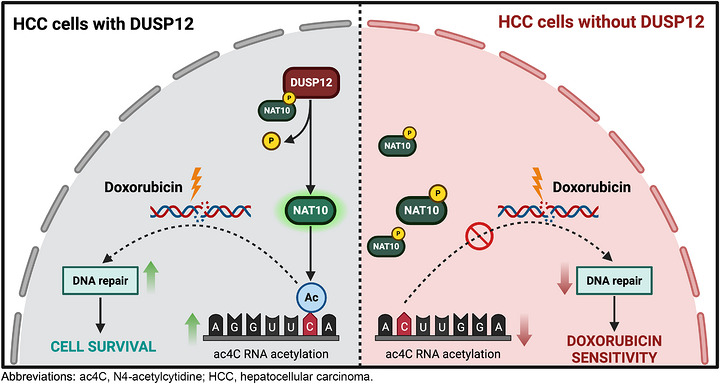

## 1 Introduction

Hepatocellular carcinoma (HCC) is the most prevalent primary liver malignancy, accounting for over 906,000 diagnosed cases globally in 2020. HCC is the third leading cause of cancer‐related mortality worldwide, with a relative 5‐year survival rate of approximately 18% [1]. Its aggressive nature and poor prognosis are primarily attributed to late‐stage diagnosis and the limited efficacy of available therapeutic options, which depend largely on combinations of tyrosine kinase inhibitors (TKIs) and immune checkpoint inhibitors [2]. Chemotherapy remains a key therapeutic strategy for HCC, including its use in transarterial chemoembolization (TACE) with agents such as doxorubicin (DX) and cisplatin (CP), which exert anticancer effects by inducing DNA damage and activating the DNA damage response (DDR)—a highly coordinated signaling network that detects DNA lesions, induces cell cycle arrest, and promotes repair to maintain genomic stability [3]. Notably, evidence suggests a functional interplay between the DDR and ribosome biogenesis (RB) pathways, highlighting their potential relevance in cancer pathophysiology.

RB is a complex and energy‐intensive process involving the synthesis, processing, and assembly of ribosomal RNA (rRNA) and ribosomal proteins [4]. The nucleolus, beyond its traditional role in ribosome production, acts as a central hub for sensing cellular stress, including DNA damage [5, 6]. Disruption of nucleolar function, often termed “nucleolar stress,” activates DDR by triggering the release of key nucleolar components, such as ribosomal proteins, which can stabilize p53 and other DDR effectors [7, 8]. Conversely, several DDR‐related proteins can impair RB [9, 10, 11, 12]. In cancer, the interplay between DDR and RB is particularly significant since many oncogenic signaling pathways involve overactivation of rRNA synthesis leading to uncontrolled cell proliferation [13, 14]. However, these changes also increase sensitivity to nucleolar stress and DNA damage, creating potential therapeutic vulnerabilities. For instance, targeting RB via nucleolar disruption has been shown to sensitize chemoresistant cells to genotoxic therapies [15, 16, 17].

The dual‐specificity phosphatase 12 (DUSP12), a relatively unexplored phosphatase in humans, seems to be implicated in both genotoxic stress response and nucleolar function. DUSP12 displays a distinctive and conserved zinc‐binding domain which points to the possibility of interactions with nucleic acids and ribonucleoproteins [18, 19]. Like its ortholog Yvh1 in yeast, DUSP12 is now recognized for its involvement in late stages of RB [20, 21, 22, 23]. Moreover, through dephosphorylation of its sole known substrate, ASK1, and subsequent attenuation of the JNK/p38 MAPK pathway, DUSP12 expression and activity are critical for regulating hepatic lipid metabolism and ensuring cell survival under conditions such as diabetic cardiomyopathy, hepatic and neuronal ischemia–reperfusion [24, 25, 26, 27]. Interestingly, DUSP12 physically interacts with nucleolar proteins under genotoxic stress conditions, such as *N*‐acetyltransferase 10 (NAT10), which is a regulator of rRNA transcription and processing [28]. NAT10 is the only enzyme identified in humans as a writer of N4‐acetylcytidine (ac4C) across a broad range of RNAs: the acetylation of mRNA and tRNA enhances translational efficiency and fidelity [29, 30]. In addition to acetylating 18S rRNA and promoting its maturation, NAT10 contributes to RB by acetylating UBF, thereby facilitating RNA Pol I recruitment to ribosomal DNA (rDNA) promoter regions [31, 32]. Through acetylation of protein substrates, NAT10 is also implicated in cellular processes such as cell division, autophagy, chromatin remodeling, and DDR [33, 34, 35, 36]. Interestingly, NAT10 is upregulated and related to progression, metastasis, and chemoresistance of HCC [37, 38, 39].

The interaction between DUSP12 and NAT10 suggests this axis may serve as a molecular bridge linking DNA damage and nucleolar stress pathways involving nucleolar proteins [28]. Importantly, while NAT10‐mediated RNA acetylation has recently emerged as a regulator of RB, translation, and DNA repair, the upstream mechanisms controlling NAT10 activity remain poorly understood. At the same time, although DUSP12 has been implicated in RB and cellular stress adaptation, its downstream molecular targets and regulatory networks are still largely undefined in mammalian systems. Therefore, linking DUSP12 to NAT10‐dependent ac4C RNA modification provides a potential mechanistic framework connecting phosphatase activity to RNA modification pathways involved in nucleolar function and genome maintenance. Thus, this study aimed to investigate this interplay in liver cells, more specifically HCC, focusing on the involvement of the DUSP12–NAT10 axis in these processes. Two genotoxic agents used in chemotherapy were evaluated in two‐ and three‐dimensional cultures of two hepatic cell lines. The role of DUSP12 was further investigated using DUSP12‐deficient HuH‐7 cells. Our findings showed that DUSP12 loss increased sensitivity to genotoxic stress and was associated with enhanced DDR activation, delayed DNA repair, and altered nucleolar organization.

## 2 Results

### 2.1 DX Is More Cytotoxic Than CP in Monolayers of Hepatic Cells

**FIGURE 1:**
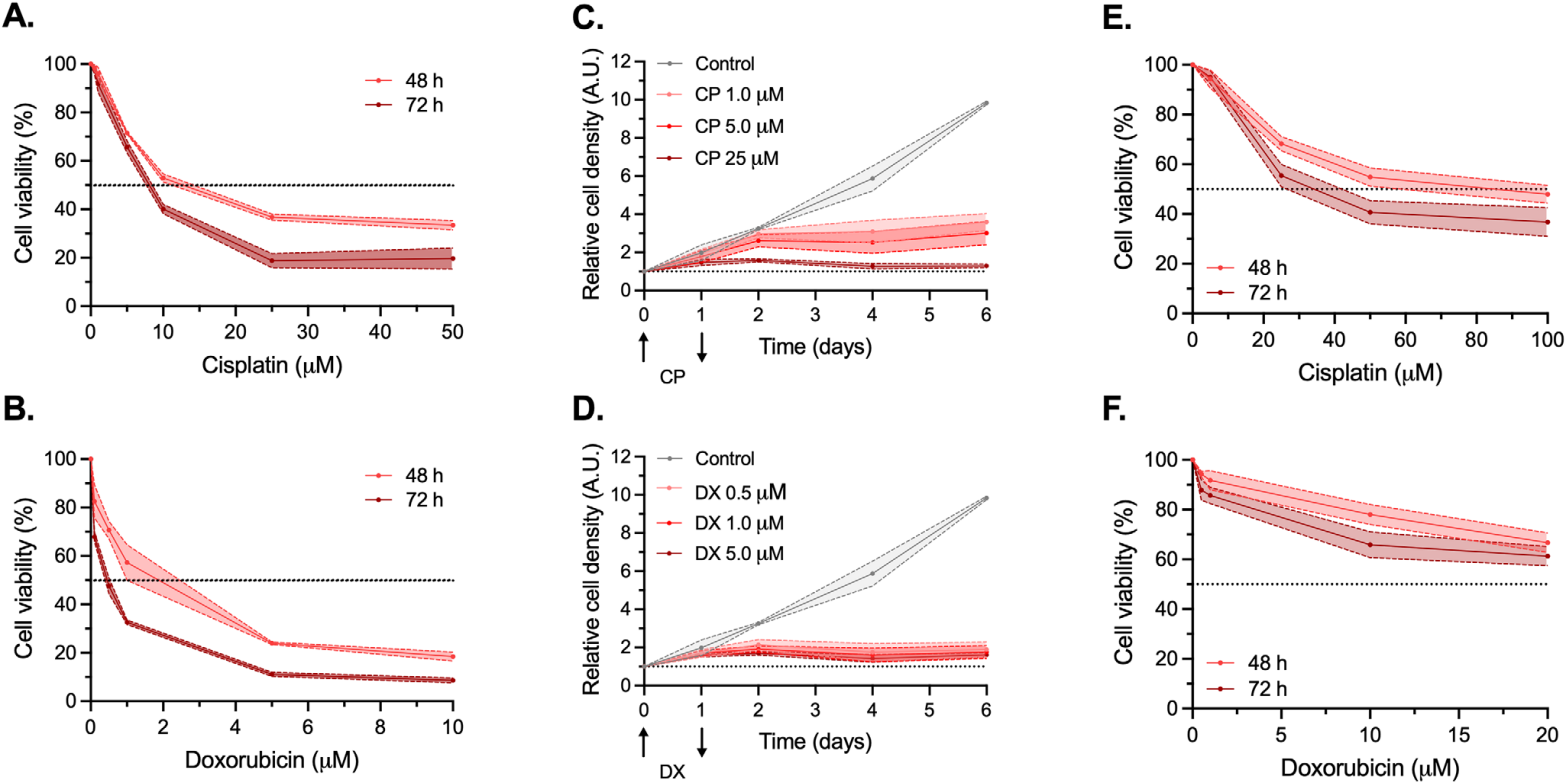
Cytotoxic effects of CP and DX in monolayers and spheroids of HuH‐7 cells. (A and B) Cell viability of HuH‐7 monolayers treated with increasing concentrations of (A) CP or (B) DX for 48 or 72 h. (C and D) Proliferation curves of HuH‐7 monolayers treated with (C) CP or (D) DX for 24 h. (E and F) Cell viability of HuH‐7 spheroids treated with increasing concentrations of (E) CP or (F) DX for 48 or 72 h. Data represent mean ± SD of at least three independent experiments. CP, cisplatin; DX, doxorubicin; SD, standard deviation.

The human HCC cell line HuH‐7 was treated with different genotoxic agents to assess whether cellular sensitivity depended on the type of DNA damage induced. Both CP and DX reduced cell viability, but DX exerted a more pronounced effect on proliferation inhibition (Figure 1A–D, Tables S4 and S5). Similar results were obtained for the hepatic progenitor cell line HepaRG (Figure S1A–D). To mimic physiological conditions, both treatments were tested in spheroids. Standardization of cell number and culture duration enabled the generation of spheroids with physiologically relevant diameters (> 350 µm) in both HuH‐7 (Figure S2A,B) and HepaRG lines (Figure S2D,E). Although spheroid diameter remained constant or even decreased under certain conditions, the acid phosphatase assay (APH) revealed an increase in the population of viable cells, suggesting that these changes reflect spheroid reorganization rather than inhibition of proliferation (Figure S2C,F). Compared to monolayers, HuH‐7 spheroids were significantly less responsive to CP and DX treatments (Figure 1E,F and Table S6). DX treatment resulted in a 150‐fold increase in IC_50_ for spheroids versus monolayers (IC_50_ monolayer, 72 h = 0.351 µM; IC_50_ spheroid, 72 h = 52.9 µM). Similar results were observed for HepaRG spheroids (Figure S1E,F). Together, these results show that DX was more cytotoxic than CP, whereas three‐dimensional growth markedly reduced the response to both agents.

### 2.2 DX Induces Stronger DDR Activation, Nucleolar Stress Features, NAT10 Translocation, and Enhanced Colocalization With DUSP12

**FIGURE 2:**
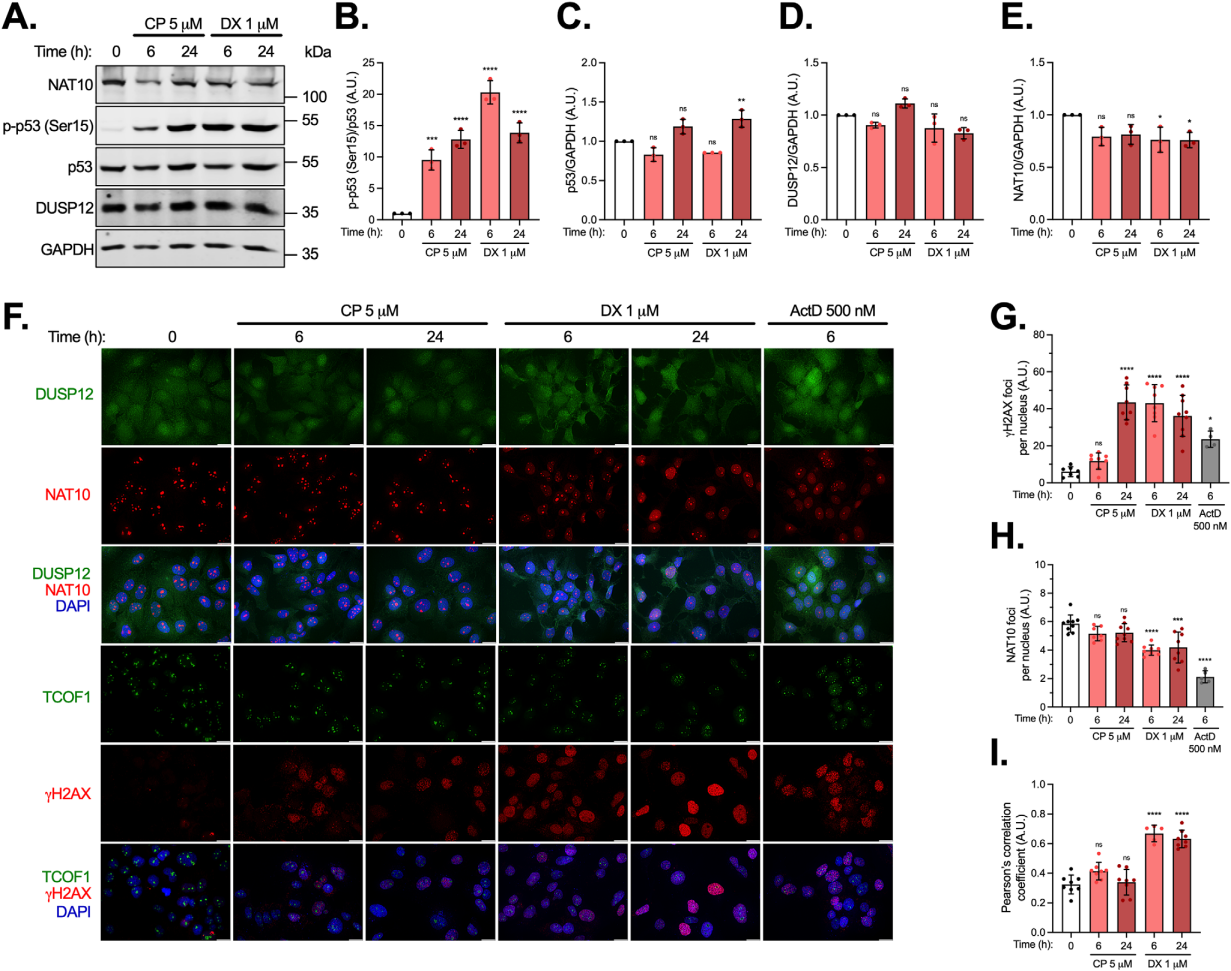
DX triggers stronger DDR activation and NAT10 relocation in HuH‐7 cells. (A) Western blot detection of NAT10, p‐p53 (also called phospho‐p53) (Ser15), total p53, DUSP12, and GAPDH in HuH‐7 cells treated with CP or DX. (B–E) Densitometric quantification of (B) p‐p53 (Ser15), (C) total p53, (D) DUSP12, and (E) NAT10 relative to GAPDH expression. (F) Representative immunofluorescence images of DUSP12, NAT10, TCOF1, and γH2AX in HuH‐7 cells treated with CP, DX, or ActD. Scale bar = 20 µm. (G) Quantification of γH2AX foci per nucleus. (H) Quantification of NAT10 foci per nucleus. (I) Colocalization of DUSP12 and NAT10 assessed by Pearson's correlation coefficient. Data represent mean ± SD of at least three independent experiments. Statistical significance relative to untreated cells is indicated as ^*^ *p* < 0.05; ^**^ *p* < 0.01; ^***^ *p* < 0.001; ^****^ *p* < 0.0001; ns = not significant. ActD, actinomycin D; CP, cisplatin; DDR, DNA damage response; DX, doxorubicin; p‐p53, phosphorylated p53; SD, standard deviation.

As evidenced by increased levels of phospho‐p53 (Ser15) and quantification of γH2AX foci, DX induced a stronger and earlier DDR activation compared to CP in both HuH‐7 (Figure 2A,B,F,G) and HepaRG cells (Figure S3A,B,F,G). The expression and phosphorylation of p53 varied according to the mutational status of the protein in each cell line (Figure 2A–C and Figure S3A–C). DUSP12 expression remained unchanged upon treatment, whereas DX induced a moderate reduction in NAT10 expression in both models (Figure 2A,D,E and Figure S3A,D,E). Subcellular distribution of DUSP12 between cytoplasmic and nuclear compartments was not affected by either treatment, as shown by immunofluorescence (Figure 2F and Figure S3F) and fractionation assays (Figure S4). Interestingly, in HepaRG cells, DUSP12 was predominantly nuclear, whereas this distribution pattern was lost in HuH‐7 cells. In contrast, NAT10 localization was markedly altered in response to DX, with translocation from the nucleolus to the nucleoplasm (Figure 2F and Figure S3F). This NAT10 redistribution was accompanied by alterations in the number of nucleoli per nucleus (Figure 2H and Figure S3H), resembling nucleolar stress, a hypothesis supported by the nucleoplasm accumulation of NAT10 upon actinomycin D (ActD) treatment, a known RNA Pol I inhibitor. ActD treatment also led to the formation of γH2AX foci, reinforcing the notion of a functional crosstalk between DDR and nucleolar stress pathways (Figure 2F,G and Figure S3F,G). Similar changes were detected for the nucleolar protein TCOF1, a validated DUSP12 interactor under genotoxic stress in tumor cells [28]. Pearson's correlation analysis of microscopy images revealed enhanced colocalization of DUSP12 and NAT10 upon DX treatment in both cell lines (Figure 2I, Figure S3I). Collectively, DX induced stronger DDR activation and more pronounced nucleolar remodeling than CP in both hepatic cell models.

### 2.3 DUSP12 Knockout Sensitizes HCC Cells and Spheroids to DX

**FIGURE 3:**
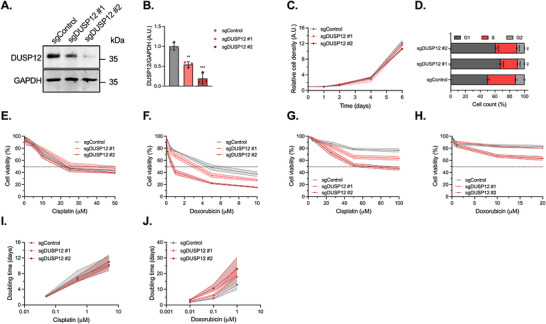
CRISPR‐mediated KO of DUSP12 sensitizes HuH‐7 cells to DX. (A) Immunoblot validation of DUSP12‐KO in HuH‐7 cells using two different sgRNAs (sgDUSP12 #1 and #2). (B) Densitometric quantification of DUSP12 relative to GAPDH expression. (C) Proliferation curves of sgControl and sgDUSP12 HuH‐7 clones in monolayer cultures. (D) Cell cycle distribution of DUSP12‐KO HuH‐7 clones. Statistical significance relative to sgControl is indicated as ^**^ *p* < 0.01; ^***^ *p* < 0.001; ns = not significant. (E and F) Cell viability of DUSP12‐KO HuH‐7 clones in monolayer cultures treated with increasing concentrations of (E) CP or (F) DX for 48 h. (G and H) Cell viability of DUSP12‐KO HuH‐7 spheroids treated with increasing concentrations of (G) CP or (H) DX for 48 or 72 h. (I and J) Doubling time of DUSP12‐KO HuH‐7 clones in monolayer cultures treated with increasing concentrations of (I) CP or (J) DX for 24 h. Graphs represent mean ± SD of at least three independent experiments. CP, cisplatin; DX, doxorubicin; KO, knockout; SD, standard deviation; sgRNA, single‐guide RNA.

To further investigate the role of DUSP12 in HCC, CRISPR‐Cas9 was employed to generate knockout (KO) clones with varying degrees of DUSP12 loss (Figure 3A,B). No changes were observed in the ability of DUSP12‐KO cells to form spheroids, although the resulting spheroids were consistently smaller in size (Figure S5A–D). Similarly, proliferation rates in both monolayer and spheroid cultures remained largely unaffected (Figure 3C, Figure S6A). DUSP12‐deficient cells exhibited impaired S‐phase entry, as indicated by G1 accumulation (Figure 3D). In monolayer cultures, DUSP12 loss did not alter CP responsiveness, but markedly increased sensitivity to DX in a manner proportional to DUSP12 expression levels (Figure 3E,F and Table S7). Notably, DUSP12‐KO spheroids (specifically sgDUSP12 #2) were more sensitive to both CP and DX (Figure 3G,H and Figure S6B,C, Table S8). Similarly, proliferation curves showed that while doubling time remained unchanged after CP treatment, DX‐treated KO cells displayed a trend of increasing doubling time with reduced DUSP12 expression (Figure 3I,J and Figure S6D–I, Table S9). These findings indicate that reduced DUSP12 expression preferentially increases HuH‐7 sensitivity to DX, particularly under three‐dimensional culture conditions.

### 2.4 DUSP12 Loss Sensitizes HuH‐7 Cells to DX Through Impaired DNA Repair and Altered Nucleolar Stress Responses

**FIGURE 4:**
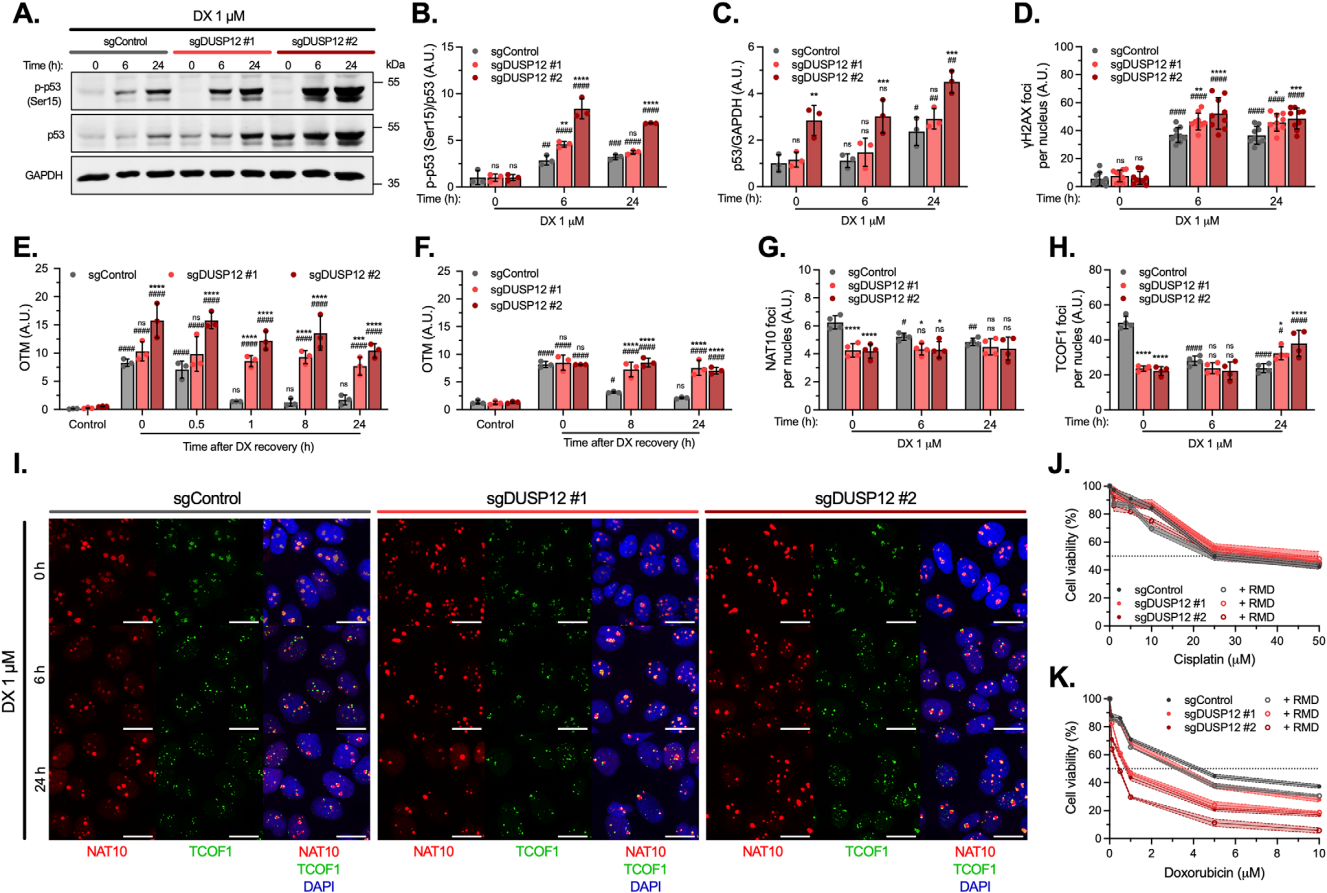
DUSP12‐KO clones exhibit enhanced DDR activation, impaired DNA repair, and nucleolar stress features after genotoxic stress. (A) Western blot detection of p‐p53 (Ser15), total p53, and GAPDH in DUSP12‐KO HuH‐7 clones treated with DX under the indicated conditions. (B and C) Densitometric quantification of (B) p‐p53 (Ser15) and (C) total p53 relative to GAPDH expression. (D) Quantification of γH2AX foci per nucleus. (E and F) DNA repair capacity assessed by Olive Tail Moment using (E) alkaline and (F) neutral comet assays in DUSP12‐KO HuH‐7 clones after 24 h of DX treatment and subsequent recovery at the indicated times. (G) Quantification of NAT10 foci per nucleus. (H) Quantification of TCOF1 foci per nucleus. (I) Representative immunofluorescence images of NAT10 and TCOF1 in DUSP12‐KO HuH‐7 clones treated with DX. Scale bar = 5 µm. (J and K) Cell viability of DUSP12‐KO HuH‐7 clones pre‐treated with 10 µM RMD for 24 h prior to exposure to increasing concentrations of (J) CP or (K) DX for 48 h (in the presence of RMD). Graphs represent mean ± SD of at least three independent experiments. Statistical significance relative to sgControl at each time point is indicated as ^*^ *p* < 0.05; ^**^ *p* < 0.01; ^***^ *p* < 0.001; ^****^ *p* < 0.0001; ns = not significant. Statistical significance relative to untreated cells is indicated below as ^#^ *p* < 0.05; ^##^ *p* < 0.01; ^###^ *p* < 0.001; ^####^ *p* < 0.0001; ns = not significant. CP, cisplatin; DDR, DNA damage response; DX, doxorubicin; KO, knockout; p‐p53, phosphorylated p53; SD, standard deviation.

To elucidate the mechanism underlying the increased sensitivity to DX observed in DUSP12‐KO cells, we first examined the two primary modes of action of DX: generation of reactive oxygen species (ROS) and induction of DNA damage. While no differences in ROS levels were detected across a wide range of DX concentrations between DUSP12‐proficient and ‐deficient clones (Figure S7A), a notably stronger DDR activation was observed in the KO clones, as evidenced by increased p53 expression and phosphorylation (Figure 4A–C) and by the quantification of γH2AX foci (Figure 4D and Figure S7B). The pronounced DDR activation observed following DX treatment appears to be a consequence of reduced repair capacity or delayed repair kinetics of both double‐strand and single‐strand DNA breaks in DUSP12‐KO cells, as demonstrated by neutral and alkaline comet assays, respectively (Figure 4E,F). Interestingly, DUSP12‐KO cells display features consistent with nucleolar stress, such as elevated basal levels of total p53 (Figure 4A,C) and fewer nucleoli per nucleus, as demonstrated by both NAT10 and TCOF1 staining (Figure 4G–I). Upon DX treatment, DUSP12‐proficient cells exhibited pronounced nucleolar remodeling characterized by NAT10 and TCOF1 redistribution from nucleoli to the nucleoplasm, leading to a marked reduction in nucleolar number and formation of nucleolar caps (Figure 4I). In contrast, DUSP12‐deficient cells displayed altered nucleolar structure and dynamics: although NAT10 redistributed to the nucleoplasm, nucleolar number remained largely unchanged, and TCOF1 was strongly displaced to the nucleoplasm, losing its spatial association with NAT10‐positive nucleoli (Figure 4G–I). Given the altered nucleolar dynamics observed in DUSP12‐deficient clones, we tested whether pharmacological inhibition of NAT10 with remodelin (RMD) could further enhance their sensitivity to genotoxic agents. Consistent with this hypothesis, RMD treatment did not sensitize cells to CP but markedly increased sensitivity to DX (Figure 4J,K). Although a modest effect was also observed in DUSP12‐proficient cells, the reduction in IC_50_ values was considerably more pronounced in DUSP12‐KO clones (Table S10). Moreover, all clones displayed comparable sensitivity to RMD alone (Figure S7C and Table S11), suggesting that the combination of DUSP12 loss and pharmacological NAT10 inhibition enhances the cellular response to DX‐induced genotoxic stress. Together, DUSP12 loss was associated with delayed DNA break repair and altered nucleolar responses, while RMD treatment further increased DX sensitivity.

### 2.5 DUSP12–NAT10 Interaction Is Associated With NAT10 Phosphorylation and RNA Acetylation

**FIGURE 5:**
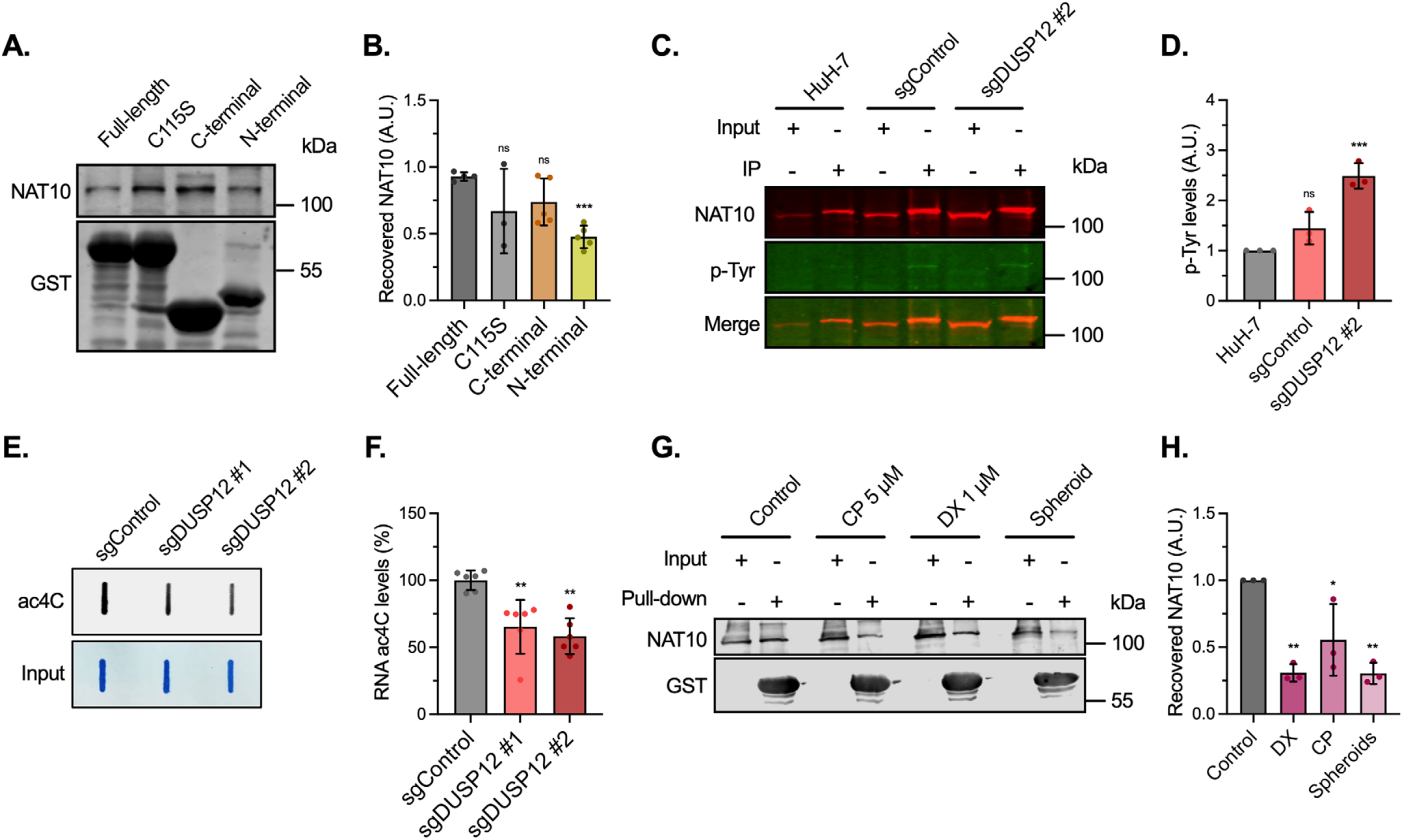
DUSP12 interaction with NAT10 is associated with changes in NAT10 phosphorylation and RNA acetylation. (A) Western blot detection of NAT10 and GST after pull‐down assays with GST‐tagged DUSP12 constructs. (B) Densitometric quantification of NAT10 binding levels. Differences relative to full‐length DUSP12 are indicated. (C) Western blot detection of NAT10 and p‐Tyr after immunoprecipitation of NAT10 in HuH‐7 parental cells, sgControl, and sgDUSP12 #2 clones. (D) Densitometric quantification of p‐Tyr levels. Differences relative to parental HuH‐7 cells are indicated. (E) Slot blot detection of ac4C in DUSP12‐KO clones. (F) Densitometric quantification of ac4C levels. Differences relative to sgControl are indicated. (G) Western blot detection of NAT10 from pull‐down assays using full‐length DUSP12 as bait incubated with lysates from HuH‐7 cells in monolayer untreated or treated with 5 µM CP or 1 µM DX for 24 h, and from HuH‐7 spheroid cultures. (H) Densitometric quantification of NAT10 binding levels. Differences relative to HuH‐7 cells in monolayer are indicated. Graphs represent mean ± SD of at least three independent experiments. Statistical significance is indicated as ^*^ *p* < 0.05; ^**^ *p* < 0.01; ^***^ *p* < 0.001; ns = not significant. ac4C, N4‐acetylcytidine; CP, cisplatin; DX, doxorubicin; KO, knockout; p‐Tyr, phosphorylated tyrosine; SD, standard deviation.

To gain insights into the interaction between DUSP12 and NAT10, pull‐down assays were performed using recombinant GST‐tagged DUSP12 constructs [28]. The full‐length and the substrate‐trapping mutant C115S displayed comparable binding to NAT10; in contrast, binding was significantly reduced when using the isolated N‐terminal domain, showing that removal of the C‐terminal zinc‐binding domain compromises the interaction, although the N‐terminal domain alone retains partial binding capacity (Figure 5A,B). To assess whether DUSP12 catalytic activity contributes to complex formation, pull‐down assays were performed in the presence of the tyrosine phosphatase inhibitor Na_3_VO_4_. After establishing the optimal conditions for DUSP12 inhibition by monitoring dephosphorylation of the general substrate DiFMUP (6,8‐difluoro‐4‐methylumbelliferyl phosphate) (Figure S8A), pull‐down assays revealed that the interaction with both the full‐length and N‐terminal constructs was significantly reduced, supporting a contribution of DUSP12 catalytic activity to efficient DUSP12–NAT10 complex formation, while inhibition had no apparent effect on binding to the isolated C‐terminal domain (Figure S8B,C). Interestingly, treatment with RMD selectively reduced binding to full‐length DUSP12 without affecting interaction with isolated domains (Figure S8D,E), suggesting that both domains—or their interface—are required for interaction with NAT10. These findings are consistent with a potentially reciprocal relationship in which phosphatase inhibition and RMD treatment influence DUSP12–NAT10 complex formation. Consistently, NAT10 immunoprecipitation followed by phosphotyrosine detection revealed increased NAT10 phosphorylation in sgDUSP12 #2 cells, supporting a role for DUSP12 in regulating NAT10 tyrosine phosphorylation under basal conditions (Figure 5C,D). Loss or reduction of DUSP12 also led to decreased levels of acetylated cytidine (ac4C) in RNA, consistent with reduced NAT10‐dependent RNA acetylation (Figure 5E,F). Finally, interaction between DUSP12 and NAT10 was reduced in spheroids and under genotoxic stress (CP or DX), suggesting stress‐induced post‐translational or conformational changes in NAT10 that impair complex formation (Figure 5G,H). Overall, these findings support an association between DUSP12–NAT10 complex formation, NAT10 phosphorylation status, and cellular ac4C levels.

### 2.6 *DUSP12* Amplification Is Frequent, and Elevated Expression Correlates With Poor Prognosis and Enrichment of RB and DDR Pathways in HCC Patients

**FIGURE 6:**
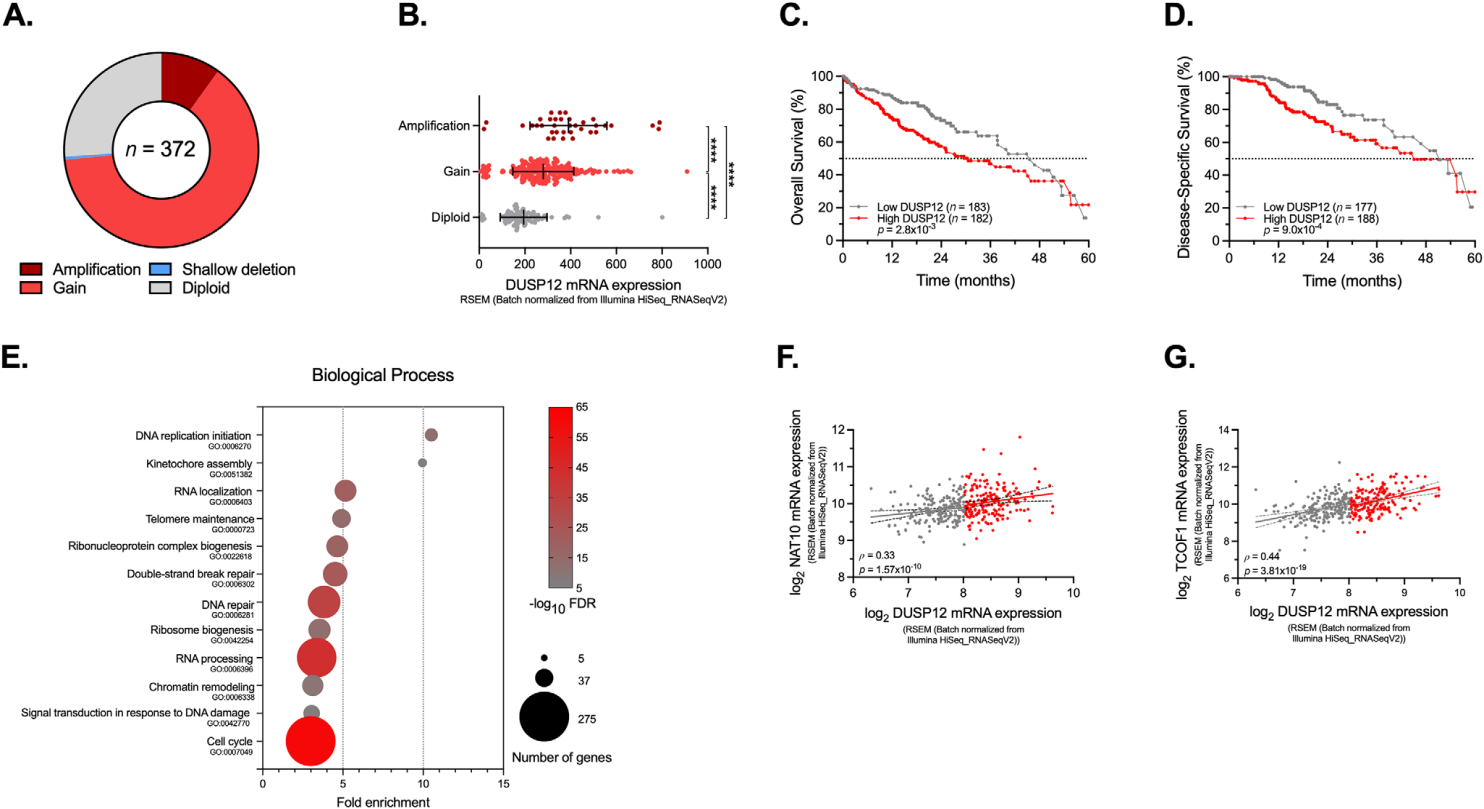
Genomic alterations and clinical significance of DUSP12 in HCC patient data. (A) Distribution of *DUSP12* copy number alterations in HCC patients. (B) DUSP12 mRNA expression in HCC patients with or without copy number alterations. (C and D) 5‐year Kaplan–Meier analyses of (C) overall survival and (D) disease‐specific survival in HCC patients stratified by median DUSP12 expression. (E) Gene Ontology analysis of biological processes associated with genes correlated with DUSP12 expression in HCC patients. (F and G) Correlation between DUSP12 expression and (F) NAT10 or (G) TCOF1 expression in HCC patients with high (red) or low (gray) DUSP12 expression. Spearman's correlation coefficient (*ρ*) and associated *p*‐value are shown. Statistical significance is indicated as ^****^ *p* < 0.0001; ns = not significant. HCC, hepatocellular carcinoma; mRNA, messenger RNA.

To examine the clinical relevance of DUSP12 in HCC, patient‐derived datasets were analyzed. The *DUSP12* gene was frequently gained (10%) or amplified (63%) in HCC cases (Figure 6A). As expected, *DUSP12* copy number gain was correlated with aneuploidy score (Figure S9A) and tended to increase with tumor grade (Figure S9B). Copy number alterations corresponded to elevated DUSP12 expression (Figure 6B), which was associated with reduced overall and disease‐specific survival (Figure 6C,D), but not with disease progression or recurrence (Figure S9C,D). Genes strongly co‐expressed with DUSP12 (*n* = 1189, *ρ* > 0.3) were enriched in pathways related to both canonical functions of DUSP12, such as ribosome and ribonucleoprotein complex biogenesis, and non‐canonical roles, including DNA replication initiation, kinetochore assembly, telomere maintenance, DNA repair, and chromatin remodeling (Figure 6E). Consistently, enrichment was also observed for cellular components such as the nucleoplasm, kinetochore, and heterochromatin, as well as molecular functions related to nucleic acid interactions (Figure S9E,F). Only 1.1% of co‐expressed genes were located near the *DUSP12* locus (cytogenetic band 1q23.3), suggesting that co‐expression reflected functional rather than positional effects (Figure S9G). Notably, 71 out of the 400 nuclear interaction partners of DUSP12 identified under genotoxic stress in our laboratory [28] were also found among the co‐expressed genes, including NAT10 and TCOF1 (Figure 6F,G and Figure S9H). Thus, elevated DUSP12 expression is associated with adverse clinical outcomes and with transcriptional programs related to RB and genome maintenance in HCC.

## 3 Discussion

In this study, we identified DUSP12 as a modulator of the cellular response to genotoxic and nucleolar stress in hepatic cancer models, uncovering a previously unrecognized regulatory axis linking an atypical dual‐specificity phosphatase to NAT10‐mediated RNA acetylation. By connecting tyrosine dephosphorylation signaling to ac4C‐dependent RNA modification, this work provides a conceptual framework integrating protein phosphorylation signaling, nucleolar homeostasis, and DDR pathways. Our data show that both CP and DX exert antiproliferative effects in models of HuH‐7 and HepaRG liver cells, with spheroid models displaying marked resistance compared to monolayers and DX being the most cytotoxic. DX treatment induced a more robust and earlier DDR than CP, evidenced by increased p53 phosphorylation and γH2AX foci formation. Interestingly, while DUSP12 expression remained stable under genotoxic stress, NAT10 levels were moderately reduced and redistributed from the nucleolus to the nucleoplasm. This relocation, the substantial alterations in nucleolar morphology and parallel changes in TCOF1 location, is collectively consistent with nucleolar stress conditions. Supporting this hypothesis, treatment with ActD, a canonical RNA Pol I inhibitor, not only promoted nucleolar NAT10 redistribution, but also triggered γH2AX foci formation, suggesting a functional crosstalk between DDR and nucleolar stress pathways. Notably, DX‐induced nucleolar stress may be particularly evident due to the preferential intercalation of DX into GC‐rich rDNA regions, thereby impairing replication and repair processes at these sites [40]. Our comparative analysis further revealed that DUSP12 localization differs between non‐tumorigenic and malignant cells: it is predominantly nuclear in HepaRG, but not in HuH‐7. This raises the possibility that subcellular distribution of DUSP12 is altered during malignant transformation, a hypothesis that requires further validation in additional models.

Previous work from our group has already shown that DUSP12 undergoes marked shifts in its interactome under different genotoxic and oxidative stress conditions (UVB, ionizing radiation, and bleomycin) in breast and lung cancer cells, suggesting its broad involvement in cellular response pathways [28]. Elevated DUSP12 expression has been observed in patients with HCC, although the functional implications of this upregulation remain poorly understood [41]. Here, loss‐of‐function experiments established a functional interplay between DUSP12 and chemoresistance. DUSP12‐KO sensitized HuH‐7 cells (in monolayer and spheroids) to DX without affecting ROS levels, pointing to a DDR‐related mechanism. Previous studies have also implicated DUSP12 in promoting cell survival under oxidative stress, potentially through the redox regulation of cysteine residues on its C‐terminal domain [27, 42, 43]. Our findings complement this model by demonstrating that DUSP12 also modulates genotoxic stress responses, thereby contributing to DX chemoresistance. Despite incomplete KO and residual expression—potentially explained by the high aneuploidy of HuH‐7 cells [44] and the frequent *DUSP12* copy number gains observed in HCC—DUSP12‐deficient cells exhibited enhanced DDR activation, likely due to a delayed or inefficient repair of both double‐ and single‐strand DNA breaks. The marked G1 arrest in DUSP12‐KO cells suggests a reduced opportunity for homologous recombination (HR), possibly shifting DNA repair toward the more error‐prone non‐homologous end joining (NHEJ) pathway. This shift could partly explain the increased sensitivity to DX that we observe, although further investigation is needed to directly validate this mechanism. It remains to be determined whether this attenuation is specific to DX‐induced double‐strand breaks and the repair pathways they engage, or whether it extends to other DNA damage contexts.

Beyond its role in DDR modulation, our findings reveal that loss of DUSP12 elicits features consistent with nucleolar stress, including increased basal p53 levels and altered nucleolar organization and dynamics. This phenotype aligns with the function of DUSP12 in RB and the evidence—both from our group and others—that DUSP12 physically interacts with numerous nucleolar and RB regulators under basal and stress conditions [18, 20, 21, 22, 23, 28]. Notably, DUSP12‐KO disrupted the localization pattern of two validated interaction partners, NAT10 and TCOF1, both of which were redistributed from nucleoli to the nucleoplasm even in the absence of genotoxic challenge and displayed further mislocalization following DX treatment. This result is particularly relevant given that NAT10 and TCOF1 are frequently upregulated in HCC and have been associated with therapeutic resistance [37, 45]. In recent years, TCOF1 has been proposed as a central coordinator of nucleolar DDR by scaffolding key signaling proteins. Mechanistically, TCOF1 recruits the ATR activator TOPBP1 in the nucleoli in an ATM‐ and NBS1‐dependent manner following rDNA breaks, which is essential for the inhibition of rRNA synthesis and nucleolar remodeling in response to damage [10, 46, 47]. Therefore, the pronounced redistribution of TCOF1 observed in DUSP12‐deficient cells may reflect impaired DDR coordination within the nucleolus and defective stress adaptation. Importantly, this nucleolar stress state appears therapeutically exploitable, as DUSP12 loss markedly sensitized cells to DX, a drug that itself induces profound nucleolar remodeling, indicating that targeting nucleolar homeostasis may represent a promising strategy to enhance chemotherapeutic efficacy in HCC.

More importantly, we described a novel molecular connection between DUSP12 and NAT10. Pull‐down assays indicated that NAT10 interacts with both the N‐ and C‐terminal regions of DUSP12, and phosphatase inhibition with Na_3_VO_4_ or pharmacological NAT10 inhibition with RMD reduced the DUSP12–NAT10 interaction. These findings are compatible with a composite interaction model in which distinct structural elements of DUSP12 contribute differentially to NAT10 binding. Because the NAT10 domains involved in this interaction were not interrogated in the present study, it remains possible that different regions of NAT10 engage different structural features of DUSP12. The sensitivity of the complex formation to both treatments suggests that DUSP12–NAT10 interaction may be functionally regulated rather than reflecting only passive colocalization or structural proximity. Importantly, the increased colocalization observed under DX treatment does not necessarily imply stronger direct binding but may instead reflect stress‐induced relocalization of both proteins to overlapping nuclear or nucleolar compartments. Conversely, the reduced pull‐down signal observed under genotoxic stress or in spheroids may result from conformational changes and/or post‐translational modifications that affect the stability of the DUSP12–NAT10 complex.

Functionally, DUSP12 loss increased NAT10 phosphotyrosine levels and impaired RNA acetylation. While ac4C is essential for maturation of 18S rRNA in several organisms, it appears dispensable for human cell survival, which may explain why DUSP12 loss does not by itself compromise proliferation [48]. Consistent with this observation, genome‐scale CRISPR dependency datasets from the Cancer Dependency Map indicate that DUSP12 is not a broadly essential gene across cancer cell lines, displaying relatively mild gene effect scores in most contexts [49, 50]. In line with these findings, genetic studies in mice have shown that DUSP12 deficiency does not cause lethality but instead exacerbates metabolic and inflammatory liver phenotypes under stress conditions such as high‐fat diet or ischemia–reperfusion injury [26, 27]. Together, these observations suggest that DUSP12 primarily contributes to cellular stress adaptation rather than core viability functions, raising the possibility that targeting the DUSP12–NAT10 axis could preferentially affect tumor cells exposed to genotoxic stress. Nevertheless, ac4C also modifies other RNAs and is being implicated in DNA repair by different mechanisms. For instance, ac4C‐mediated stabilization of AHNAK mRNA is required for recruitment of LIG4 and XRCC4 to chromatin during NHEJ, while ac4C‐mediated destabilization of DDB2 mRNA has been shown to attenuate the global genome nucleotide excision repair (GG‐NER) pathway [51, 52]. In addition, ac4C has been detected at damaged chromatin and implicated in DNA repair through both NAT10‐independent and NAT10‐dependent mechanisms. In the latter context, NAT10‐dependent ac4C modification of RNA within DNA:RNA hybrids at DSBs promotes chromatin decompaction and HR repair [53, 54]. In line with this functional connection, treatment with RMD, a widely used pharmacological inhibitor of NAT10, further enhanced DX sensitivity in DUSP12‐KO cells. Although formal drug–interaction analyses and genetic NAT10 perturbation will be required to establish the underlying mechanism, the preferential response of DUSP12‐deficient cells raises the possibility of a conditional synthetic vulnerability under DX‐induced genotoxic stress. Previous studies have reported that NAT10 knockdown sensitizes bladder cancer cells to CP [51]; however, our results do not exclude a potential interaction between DUSP12 loss and pharmacological targeting of NAT10‐associated functions under CP treatment. We therefore propose that this discrepancy may reflect cell type‐specific differences in NAT10 dependency, DNA repair capacity, or drug response.

Finally, to gain insights and validate our experimental findings, analysis of patient‐derived datasets revealed that *DUSP12* amplification is frequent in HCC, while increased DUSP12 expression correlates with NAT10 expression and poor prognosis. Moreover, DUSP12 was co‐expressed with genes enriched in RB, DDR, and chromatin‐related pathways. Notably, a recent study demonstrated that NAT10 promotes HCC progression through ac4C‐dependent stabilization of oncogenic transcripts such as DNA damage‐induced apoptosis suppressor DDIAS [55]. In addition, ac4C‐related mRNA modification patterns stratify HCC patients into prognostically distinct groups, in which higher ac4C signatures associate with more aggressive tumor features, increased DNA‐repair pathway activation, and poorer clinical outcomes, including reduced benefit from standard treatments and differential response to anti‐PD‐1 immunotherapy and mTOR inhibition [56]. These clinical observations reinforce our findings by highlighting the physiological relevance of ac4C regulation in DDRs and therapeutic sensitivity. Taken together, our observations position DUSP12 as a potential regulator of NAT10‐associated functions, linking tyrosine dephosphorylation to RNA acetylation control, and place the DUSP12–NAT10–ac4C axis at the intersection of nucleolar function, DNA repair capacity, and clinically relevant determinants of HCC prognosis and treatment response. However, this study faces difficulties and limitations that, once overcome, will further strengthen the findings reported here. First, most functional experiments were performed in two hepatic cell models, and the consequences of DUSP12 loss were examined primarily in HuH‐7‐derived clones; validation in additional HCC models and in vivo systems should therefore be considered. Second, although our data support a regulatory association between DUSP12 and NAT10, they do not confirm NAT10 as a direct DUSP12 substrate or identify the relevant phosphotyrosine residues involved. Third, ac4C was evaluated using an antibody‐based slot‐blot assay, which does not resolve transcript‐ or site‐specific changes. Finally, the effects observed with RMD cannot be attributed exclusively to NAT10 and should be confirmed using genetic NAT10 perturbation or orthogonal pharmacological approaches.

## 4 Conclusion

This study identifies DUSP12 as a modulator of the cellular response to genotoxic and nucleolar stress in HCC. DUSP12‐KO was associated with altered NAT10 phosphorylation, reduced RNA ac4C levels, delayed DNA repair kinetics, enhanced DDR signaling, and altered nucleolar organization. Our findings show that DUSP12 loss sensitizes HuH‐7 cells to DX, raising the possibility of a conditional synthetic vulnerability to pharmacological NAT10 inhibition under genotoxic stress. Collectively, these findings pose the DUSP12–NAT10–ac4C axis as a potential link among genome maintenance, nucleolar function, and stress response, supporting further investigation of DUSP12 as a potential therapeutic target in HCC.

## 5 Materials and Methods

### 5.1 Cell Culture

HepaRG is an immortalized hepatic cell line that retains many characteristics of primary human hepatocytes obtained from liver tissue of an adult female (Thermo Fisher Scientific). The HuH‐7 cell line is an immortalized, tumorigenic human liver cancer cell line established in 1982 from an HCC in a 57‐year‐old Japanese male (Sigma‐Aldrich). Both cell lines were obtained in 2022 by Prof. Dr. Natália Mayumi Inada (IFSC–USP) and kindly donated to our laboratory. Cells were maintained in DMEM (Invitrogen) supplemented with 10% fetal bovine serum (Cultilab), ampicillin (25 µg/mL), and streptomycin (100 µg/mL), incubated at 37°C in a humidified atmosphere containing 5% CO_2_, and maintained in culture only up to passage 20. HuH‐7 and HepaRG cells were authenticated by short tandem repeat (STR) profiling and were routinely confirmed to be free of mycoplasma contamination using the Myco‐Sniff Mycoplasma PCR Detection Kit (MP Biomedicals) before use in the experiments.

### 5.2 Chemical Treatments

DX (Sigma, D1515), ActD (Invitrogen, A7592), and RMD (MedChem Express, HY‐16706) were prepared as 10 mM stock solutions in DMSO, and CP (Sigma, P4394) was prepared as 10 mM in 0.9% NaCl.

### 5.3 CRISPR/Cas9‐Mediated Knockout of DUSP12

DUSP12‐KO was generated using sgRNAs cloned into the eSpCas9(1.1) vector (Addgene #71814) (sequences in Table S1). Because this vector does not contain an antibiotic resistance marker, cells were co‐transfected with pEGFP‐C1 to enable selection through its neomycin/G418 resistance cassette. Co‐transfection was performed at a 10:1 ratio of eSpCas9(1.1) to pEGFP‐C1 using Lipofectamine 3000 (Invitrogen, L3000001) according to the manufacturer's instructions. Approximately 72 h after transfection, G418 selection (Sigma, A1720, 1 mg/mL) was initiated and maintained for 21 days. Surviving resistant cells were subsequently subjected to limiting dilution to isolate single‐cell clones, which were screened for DUSP12 expression by immunoblotting.

### 5.4 Cell Viability and Proliferation Assays

Cell viability and proliferation in monolayer cultures were assessed using the MTT (Invitrogen, M6494) reduction and the crystal violet incorporation assay, respectively. For cell viability, cells were incubated with 500 µg/mL MTT for 3 h at 37°C, followed by solubilization of formazan in DMSO and absorbance measurement (570 nm) using an Infinite M200 Pro (Tecan Group) microplate reader. For cell proliferation, cells were stained with 0.5% crystal violet in 20% methanol for 10 min, followed by the incorporated crystal violet elution in 33% acetic acid and absorbance measurement (590 nm). IC_50_ values were calculated using nonlinear regression with GraphPad Prism v.10.

### 5.5 Spheroid Formation and Viability

Spheroids were generated via the liquid overlay method on 96‐well plates coated with 1.5% agarose [57]. Morphometry was performed to evaluate spheroid size, using phase‐contrast microscopy (Leica DMi1, 5X objective). Viable cell populations in intact spheroids were quantified with the APH, measuring absorbance of *p*‐nitrophenolate at 405 nm [58].

### 5.6 Immunofluorescence and Confocal Microscopy

Cells were seeded on glass coverslips, fixed with 4% PFA, and permeabilized with Triton X‐100 on ice under optimized conditions: 0.5% for 5 min for HepaRG, or 1% for 10 min for HuH‐7 and derived cell lines. Blocking was carried out in 3% BSA with 10% FBS, followed by primary antibodies (overnight, 4°C) and Alexa Fluor‐conjugated secondary antibodies (1 h, room temperature) incubations. All antibodies used are listed in Table S2. Nuclei were counterstained with DAPI, and slides were mounted with ProLong Diamond (Invitrogen, P36961). Images were acquired using a Leica DMi8 fluorescence microscope, and confocal imaging was performed with a Leica SP8 STED FALCON system.

### 5.7 Western Blotting

Cell fractionation and total cellular lysates were obtained and quantified as previously described [28, 59]. Equal amounts of protein (25–50 µg) were separated by 12% SDS‐PAGE and transferred to nitrocellulose membranes (Millipore). Membranes were blocked in 3% BSA in TBST, followed by primary antibodies (overnight, 4°C) and Alexa Fluor‐conjugated secondary antibodies (1 h, room temperature) incubations. All antibodies used are listed in Table S3. Detection was performed with the Odyssey Infrared Imaging System (LICOR Biosciences). Quantification was performed in ImageJ by densitometry and normalized by the control condition.

### 5.8 Cell Cycle Analysis

Exponentially growing cells were incubated with 10 µM BrdU (Invitrogen, B23151) for 30 min, followed by fixation and permeabilization as described previously. Acid‐wash protocol was performed according to the manufacturer's instructions. Blocking and immunodetection were performed as previously described using anti‐BrdU and anti‐geminin antibodies (Table S2). Fluorescence microscopy images were acquired on a custom TissueFAXS i‐Fluo system (TissueGnostics) mounted on a Zeiss AxioObserver 7 and an ORCA Flash 4.0 v3 camera (Hamamatsu). Image analysis was performed using StrataQuest software (TissueGnostics).

### 5.9 Comet Assay

Alkaline and neutral comet assays were performed to detect DNA fragmentation, as described previously [60] with modifications. Cells were lysed and deproteinized (2.5 M NaCl, 100 mM EDTA, 1% Triton X‐100, 10% DMSO, 10 mM Tris, pH 10), DNA was denatured, and electrophoresis was carried out in alkaline buffer (300 mM NaOH, 1 mM EDTA, pH > 13) for 30 min or in neutral buffer (300 mM NaOAc, 100 mM EDTA, pH 8.3) for 1 h, both at 300 mA and 4°C. Slides were neutralized with 400 mM Tris (pH 7.4), fixed in ethanol, stained with SYBR Green II (Invitrogen, S7564), and analyzed using Comet Assay IV (Instem) software.

### 5.10 Detection of Intracellular ROS Levels

Cytoplasmic ROS levels were measured using CellROX Deep Red (Invitrogen, C10422). Cells were seeded in black, clear‐bottom 96‐well plates, treated with CP or DX for 6 h, and then incubated for 1 h with PBS containing 2.5 µM CellROX Deep Red and 10 µM Hoechst (Invitrogen, H3570). After replacing the staining solution with fresh culture medium, fluorescence was measured using an Infinite M200 Pro (Tecan Group) microplate reader at 644/665 nm excitation/emission. Each condition was analyzed in triplicate wells.

### 5.11 RNA Extraction and Slot Blot

Total RNA was isolated using TRIzol reagent (Invitrogen, 15596026). RNA purity (A260/A280 = 1.8–2.1) was verified with a NanoDrop 2000 (Thermo Fisher Scientific) spectrophotometer, and integrity was assessed using the RNA 6000 Nano kit (Agilent, 5067‐1511) on a Bioanalyzer 2100 (Agilent) system. For ac4C detection, 500 ng of RNA was denatured at 65°C for 30 min in 1 mM EDTA (pH 8.0), 4% formaldehyde, and 10X SSC (5:2:3), then blotted onto Hybond N+ (Cytiva, RPN203B) membranes using a Bio‐dot apparatus (Bio‐Rad) and UV‐crosslinked (120 mJ/cm^2^, 1 min). Membranes were stained with methylene blue (50 µg/mL in 5% acetic acid) as loading control. Blocking and immunodetection were performed as previously described using anti‐ac4C antibody (Table S3). Detection and quantification were performed as previously described.

### 5.12 Pull‐Down and NAT10 Immunoprecipitation Assays

Plasmids to produce recombinant DUSP12 (full‐length, N‐terminal, and C‐terminal domains) were kindly provided by Prof. Vacratsis [43]. Proteins were expressed in *E. coli* BL21‐DE3 and purified from bacterial lysates using glutathione‐agarose beads (Invitrogen). For pull‐down assays, cell lysates were incubated with recombinant GST‐tagged DUSP12 constructs at a 10:1 mass ratio, as previously described [28]. To assess the effects of NAT10 inhibition on the interaction, lysates of untreated cells were pre‐incubated with RMD (0.1, 1, and 10 µM) for 30 min at 4°C prior to addition of GST‐DUSP12 constructs. Alternatively, to assess the effects of phosphatase inhibition, GST‐DUSP12 constructs were pre‐incubated with 50 mM Na_3_VO_4_ for 30 min at 4°C before the addition of lysates of untreated cells. For drug treatments, cells were exposed to DX or CP prior to lysis, and lysates were subsequently subjected to the interaction assays. Following incubation, the beads were washed and directly resolved on 12% SDS‐PAGE for subsequent detection of NAT10. Normalization was performed using a GST‐specific primary antibody (Table S3) and was further adjusted based on the total lysate input to account for global protein expression under drug‐treated conditions (DX and CP) or in spheroid cultures. For NAT10 immunoprecipitation, the Protein A/G Plus kit (Santa Cruz Biotechnology, sc‐2003) was used according to the manufacturer's instructions. Immunoprecipitated samples were analyzed using a phosphotyrosine‐specific primary antibody (Table S3), and the signal was additionally normalized to the overall NAT10 expression levels in the corresponding lysate.

### 5.13 Bioinformatics Analysis

Liver hepatocellular carcinoma (LIHC) data from TCGA (PanCancer Atlas, *n* = 372) were obtained through cBioPortal. DUSP12 co‐expressed genes were identified using Spearman's rank correlation coefficients calculated from batch‐normalized RNA‐seq expression values (Illumina HiSeq RNASeqV2 RSEM data). Genes with correlation coefficients greater than 0.3 (*ρ* > 0.3) were considered positively co‐expressed and selected for further analysis. Only samples with available expression data for both genes were included in correlation calculations [61]. Gene Ontology enrichment analysis was performed using ShinyGO version 0.81. The list of co‐expressed genes was used as input, and enrichment was calculated against the background of all human protein‐coding genes. *p*‐values were corrected for multiple testing using the Benjamini‐Hochberg false discovery rate (FDR). Representative biological processes shown in the figure were selected based on statistical significance (−log_10_ FDR > 5) and fold enrichment (> 2) [62].

### 5.14 Statistical Analysis

Statistical analyses were performed using biological replicates (*n* ≥ 3), while technical replicates were averaged within each experiment prior to analysis. Statistical comparisons were performed using one‐way or two‐way ANOVA followed by Tukey's multiple comparisons test using GraphPad Prism v.10. Differences between Kaplan–Meier survival curves were assessed using the log‐rank (Mantel–Cox) test. Statistical significance was denoted as *p* < 0.05 (^*^ or #), *p* < 0.01 (^**^ or ##), *p* < 0.001 (^***^ or ###), *p* < 0.0001 (^****^ or ####), and ns = not significant.

## Author Contributions

V.K.B. and F.L.F. conceived and designed the study. V.K.B., D.R.D.C.G.P., and F.L.F. developed the methodology. V.K.B., Y.T.M., D.R.D.C.G.P., L.L.T., I.Y.A.O., and F.L.F. acquired the data. V.K.B., Y.T.M., D.R.D.C.G.P., L.L.T., I.Y.A.O., N.C.H., and F.L.F. analyzed and interpreted the data. V.K.B., D.R.D.C.G.P., I.Y.A.O., N.C.H., and F.L.F. wrote, reviewed, and revised the manuscript. V.K.B. and F.L.F. provided administrative, technical, or material support. F.L.F. acquired funding and supervised the study. All authors have read and approved the final manuscript.

## Funding

This work was supported by grants from the São Paulo Research Foundation (FAPESP; Grant Nos. 2022/04243‐1 and 2024/13597‐0), the Coordination for the Improvement of Higher Education Personnel (CAPES; Grant No. 88887.136364/2017‐00), and the National Council for Scientific and Technological Development (CNPq; Grant Nos. 304357/2024‐3, 444397/2024‐8, and 304358/2021‐5) to Fábio Luís Forti. Viktor Kalbermatter Boell is the recipient of a FAPESP PhD fellowship (Grant No. 2022/13414‐7). The microscope used for cell cycle analysis in this study was funded by FAPESP grant 2019/06039‐2 (to Nicolas Carlos Hoch).

## Ethics Statement

This study did not involve the direct recruitment of human participants or the collection of identifiable clinical information. The clinical and genomic data analyzed were deidentified and obtained from The Cancer Genome Atlas through cBioPortal. The original data collection studies were conducted with approval from the relevant institutional review boards and with informed consent from the participants. Therefore, no additional ethical approval was required for this secondary analysis of publicly available data.

## Conflicts of Interest

The authors declare no conflicts of interest.

## Supporting information




**Supporting File**: mco270929‐sup‐0001‐SuppMat.docx

## Data Availability

The data supporting the findings of this study are available within the article and its Supporting Information. Additional data are available from the corresponding author upon reasonable request. Publicly available TCGA–LIHC data analyzed in this study can be accessed through cBioPortal.
